# Identification of Novel Targets of Knee Osteoarthritis Shared by Cartilage and Synovial Tissue

**DOI:** 10.3390/ijms21176033

**Published:** 2020-08-22

**Authors:** Chenshuang Li, Zhong Zheng

**Affiliations:** 1 Department of Orthodontics, School of Dental Medicine, University of Pennsylvania, Philadelphia, PA 19104, USA; lichens@upenn.edu; 2 Section of Orthodontics, Dental and Craniofacial Research Institute and Division of Growth and Development, School of Dentistry, University of California, Los Angeles, CA 90095, USA

**Keywords:** osteoarthritis, cartilage, synovium, whole transcriptome sequencing, biomarker

## Abstract

Arthritis is the leading cause of disability among adults, while osteoarthritis (OA) is the most common form of arthritis that results in cartilage loss. However, accumulating evidence suggests that the protective hyaline cartilage should not be the sole focus of OA treatment. Particularly, synovium also plays essential roles in OA’s initiation and progression and warrants serious consideration when battling against OA. Thus, biomarkers with similar OA-responsive expressions in cartilage and synovium should be the potential targets for OA treatment. On the other hand, molecules with a distinguished response during OA in cartilage and synovium should be ruled out as OA therapeutic(s) to avoid controversial effects in different tissues. Here, to pave the path for developing a new generation of OA therapeutics, two published transcriptome datasets of knee articular cartilage and synovium were analyzed in-depth. Genes with statistically significantly different expression in OA and healthy cartilage were compared with those in the synovium. Thirty-five genes with similar OA-responsive expression in both tissues were identified while recognizing three genes with opposite OA-responsive alteration trends in cartilage and synovium. These genes were clustered based on the currently available knowledge, and the potential impacts of these clusters in OA were explored.

## 1. Introduction

Arthritis appears in over 100 identified diseases that can damage any joint in the body, causing inflammation that results in pain, stiffness, swelling, and decreased motion [1,2]. As the leading cause of disability among adults [3], arthritis has been diagnosed in approximately 54.4 million people in the U.S. alone [2,3]. Since arthritis affects people of all ages, sex, and races, its prevalence is expected to increase sharply shortly and turns to be a tremendous economic burden on patients and society [3,4,5]. Especially, osteoarthritis (OA) is the most common form of arthritis and affects around 18% of women and 10% of men over 60 [4,6]. Alarmingly, recent studies suggest that younger adults are also suffering from OA [7] associated with trauma and occupation-related joint stress [8]. Unfortunately, there are currently no approved disease-modifying osteoarthritis drugs (DMOADs) that can prevent, stop, or even restrain the progression of OA [4,9,10]. Thus, the Osteoarthritis Research Society International (OARSI) recognizes OA as an incurable condition [4]. When considering productivity loss due to OA, estimates are between 0.25% and 0.50% of the gross domestic product (GDP) [11]. Therefore, the biomedical burden of OA is enormous, growing, and inadequately addressed.

Since OA is primarily characterized by disordered articular cartilage homeostasis with subsequent inflammation and degradation, the major effects of developing an ideal OA-combating agent were focused on protecting and reestablishing the hyaline cartilage [12]. However, due to the development of the imaging and diagnosis techniques, synovitis has also been recognized as common in OA in the past decade and offers another potential target for treatment [13]. In response to this finding, glucocorticoids and non-steroidal anti-inflammatory drugs (NSAIDs) are the most prescribed OA medications [14]. For instance, glucocorticoids, such as prednisone and cortisone, are broadly used for current arthritis treatment due to their anti-inflammatory potency [15,16,17] and achieving short-term improvement in symptoms of OA [18,19]. However, the effect may vary substantially in different patient groups [18,20]. More importantly, multiple adverse side-effects in the musculoskeletal, cardiovascular, and gastrointestinal systems [20,21,22] challenge the application of glucocorticoids as a safe treatment option. Meanwhile, NSAIDs do not adequately control OA progression [23], while their long-term usage is associated with potentially harmful adverse effects [24]. Even more disappointing, the efficacy of disease-modifying antirheumatic drugs (DMARDs) that postpone rheumatoid arthritis (RA) progression by slowing or suppressing inflammation has not been replicated in OA clinical trials via systemic or local administration [25,26,27], which may be attributed to their failure on directly managing cartilage destruction—the primary cause of OA [4,28].

Although hyaline cartilage and synovium can cross-talk via synovial fluid [14] and share some inflammatory signaling pathways [29], it is worth noting that they are two distinct types of tissues and may respond differently to the same stimulation, such as OA. Strategically, the agents benefiting one tissue with the expense of another should be avoided for OA treatment. On the other hand, the biomarker(s) has/have similar OA-responsive expressions in cartilage and synovium should be a more suitable marker for OA progression than those only altered in one of these two types of tissues. Moreover, the bioactive molecule that defends and rebuilds both hyaline cartilage and synovium is favorable for OA control and treatment with no doubt. Therefore, publicly available transcriptome datasets of knee articular cartilage and synovial tissue were integrated and analyzed in the current study to gain insight into developing the new generation of DMOADs that promote both cartilage and synovium.

## 2. Results

### 2.1. Initial Evaluation of the Synovial and Cartilage RNA-seq Data Sets

By using the keywords “osteoarthritis” in the https://www.ncbi.nlm.nih.gov/gds with the selection of “*Homo sapiens*” under the column of “Top Organisms” and “Expression profiling by high throughput sequencing” under the column of “study type”, 499 items were identified which containing 4 datasets, 72 series, and 423 samples. After reviewing all the items, one dataset (GSE114007) containing transcriptome data of human knee cartilage from 18 healthy (5 females, 13 males) and 20 OA (11 females, 9 males) samples, and one dataset (GSE89408) containing transcriptome data of human synovium from 28 healthy (14 females, 14 males) and 22 OA (13 females, 9 males) samples that cover the U.S. population were included in the current study. After removing the samples with an overall alignment rate ≤75%, there were 11 human healthy cartilage samples (4 females, 9 males), 14 human OA cartilage samples (8 females, 6 males), 22 human healthy synovium samples (10 females, 12 males) and 20 human OA synovium (12 females, 8 males) samples underwent further analysis (Table S1).

**Figure 1:**
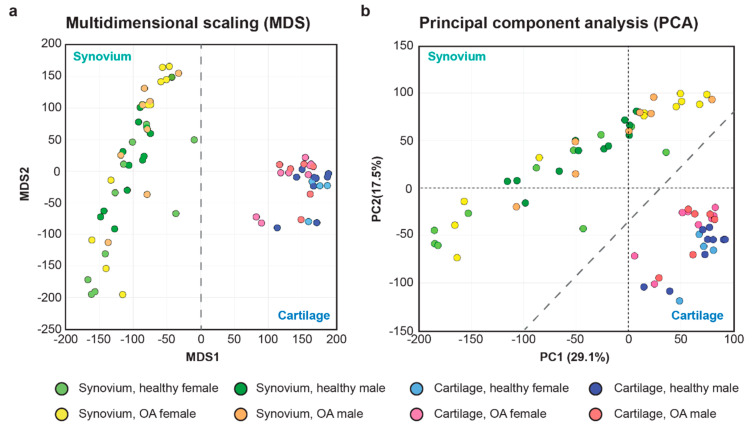
Multidimensional scaling (MDS) (**a**) and principal component analysis (PCA) (**b**) distinguish transcriptome of synovium and cartilage samples included in the current study.

By distinguishing the gene expression pattern between cartilage and synovium, multidimensional scaling (MDS) and principal component analysis (PCA) on the transcriptome profiling validated the quality of the included samples with minimum tissue contamination as expected (Figure 1).

### 2.2. Identification of the Common OA-Responsive Genes in Both Cartilage and Synovium

**Figure 2:**
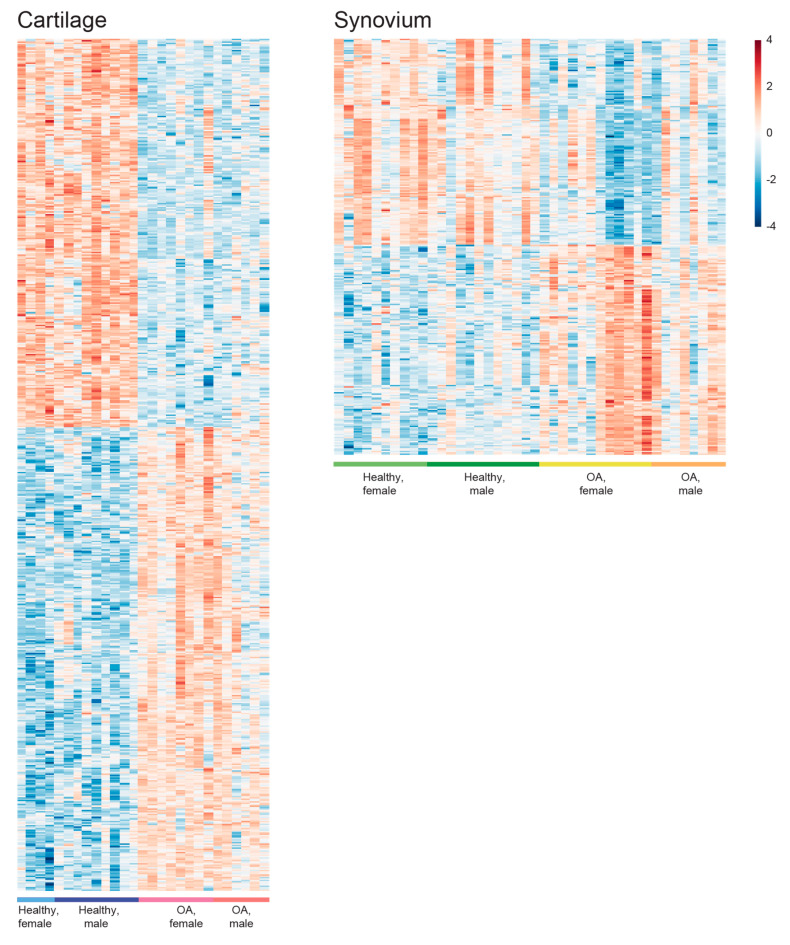
Heatmaps visualize the osteoarthritis (OA)-responsive differential expressed genes (DEGs) in cartilage and synovium, respectively.

**Figure 3:**
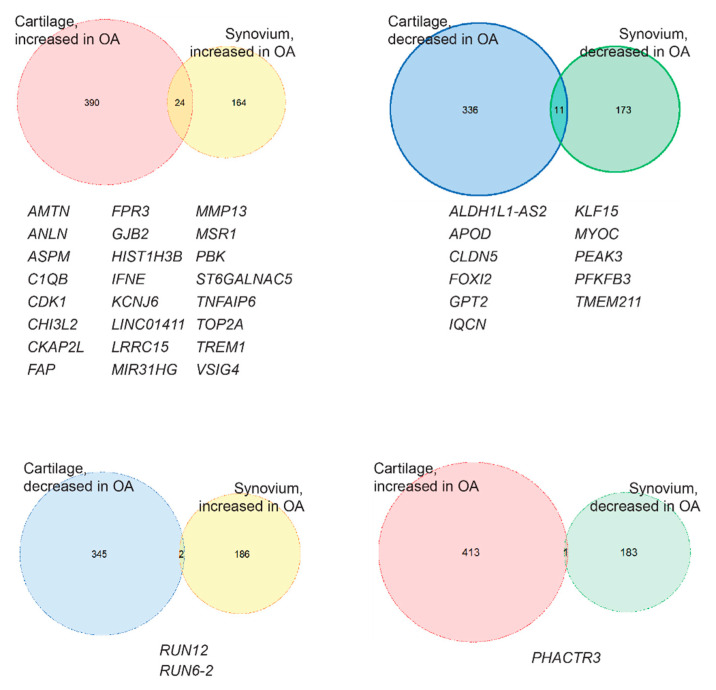
Venn diagrams visualize the osteoarthritis (OA)-responsive differential expressed genes (DEGs) with the same or different expression trends in cartilage and synovium. *AMTN*—*Amelotin*; *ANLN*—*Anillin*; *ASPM*—*Abnormal spindle-like microcephaly-associated protein*; *C1QB*—*complement C1q subcomponent subunit B*; *CDK1*—*cyclin-dependent kinase 1*; *CHI3L2*—*Chitinase-3-like protein 2*; *CKAP2L*—*cytoskeleton-associated protein 2 like*; *FAP*—*fibroblast activation protein alpha*; *FPR3*—*N-formyl peptide receptor 3*; *GJB2*—*Gap junction beta-2 protein*; *H1ST1H3B*—*histone cluster 1 H3 family member b*; *IFNE*—*Interferon epsilon*; *KCNJ6*—*G protein-activated inward rectifier potassium channel 2/Potassium voltage-gated channel subfamily J member 6*; *LINC01411*—*long intergenic non-protein coding RNA 1411*; *LRRC15*—*Leucine-rich repeat-containing protein 15*; *MIR31HG*—*MIR31 host gene*; *MMP13*—*Matrix metallopeptidase-13*; *MSR1*—*Macrophage scavenger receptor types I and II*; *PBK*—*Lymphokine-activated killer T-cell-originated protein kinase*; *ST6GALNAC5*—*Alpha-N-acetylgalactosaminide alpha-2,6-sialyltransferase 5*; *TNFAIP6*—*Tumor necrosis factor-inducible gene 6 protein*; *TOP2A*—*DNA topoisomerase 2-alpha*; *TREM1*—*Triggering receptor expressed on myeloid cell 1*; *VSIG4*—*V-set and immunoglobulin domain containing 4*; *ALDH1L1-AS2*—*ALDH1L1 antisense RNA 2*; *APOD*—*Apolipoprotein*; *CLDN5*—*Claudin-5*; *FOXI2*—*forkhead box I2*; *GPT2*—*Glutamate pyruvate transaminase 2/Alanine aminotransferase 2*; *IQCN*—*IQ motif containing N*; *KLF15*—*Krueppel-like factor 15*; *MYOC*—*Myocilin*; *PEAK3*—*PEAK family member 3*; *PFKFB3*—*6-phosphofructo-2-kinase/fructose-2,6-biphosphatase 3*; *TMEM211*—*transmembrane protein 211*; *RUN12*-*RNA U12 small nuclear*; *RUN6-2*—*RNA U6 small nuclear 2*; *PHACTR3*—*Phosphatase and actin regulator 3*.

Heatmaps of the differential expression genes (DEGs) in cartilage and synovium samples were generated, respectively, visualizing the gene expression changes between healthy and OA samples (Figure 2). In both cartilage and synovium, healthy samples could be easily distinguished from OA samples. Interestingly, cartilage displays more gene alteration in response to OA than synovium: 761 DEGs (414 increased, 347 decreased) were identified when comparing OA cartilage samples to their healthy counterparts and 372 OA-responsive DEGs (188 increased, 184 decreased) in synovium samples (Figure 2 and Figure 3). Then, by comparing the OA-responsive DEGs in cartilage and synovium, we recognized 24 upregulated and 11 downregulated genes shared by these two tissues (Figure 3). In addition, there were 3 genes that exhibited different expression trends in cartilage and synovium in OA (Figure 3).

### 2.3. Pathway Enrichment and Protein-Protein Interaction Cluster

**Figure 4:**
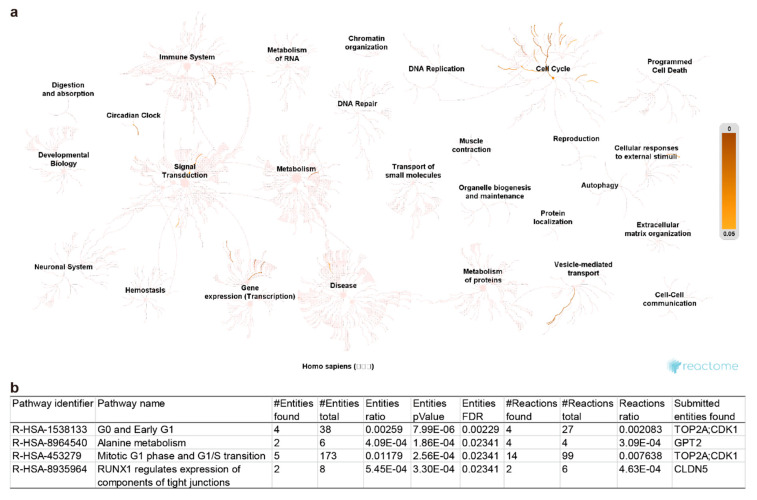
Pathway enrichment of the 35 common osteoarthritis (OA)-responsive differential expressed genes (DEGs) in cartilage and synovium has been conducted against the Reactome database. (**a**) The diagram of the distribution of the identified pathways. (**b**) The list of the identified pathways with a false discovery rate (FDR) less than 0.05. *TOP2A*—*DNA topoisomerase 2-alpha*; *CDK1*—*cyclin-dependent kinase 1*; *GPT2*—*Glutamate pyruvate transaminase 2/Alanine aminotransferase 2*; *CLDN5*—*Claudin-5*.

Firstly, pathway enrichment against the Reactome knowledgebase [30] was employed to get insight into the potential underlying regulation pathways. Nineteen of the total 35 DEGs were successfully enriched in 276 pathways among 21 clusters (Figure 4a and Table S2). Among them, 4 pathways, including “G0 and early G1” and “mitotic G1 phase and G1/S transition” pathways of the “cell cycle” cluster, “alanine metabolism” of the “metabolism” cluster, and “RUNX1 regulates expression of components of tight junctions” pathway of the “gene expression (Transcription)” cluster, display a false discovery rate (FDR) value less than 0.05 (Figure 4b), highlighting themselves as the potential vital OA-responsive pathways in both cartilage and synovium. Noticeably, 16 other DEGs, including *Leucine-rich repeat-containing protein 15* (*LRRC15*), *Myocilin* (*MYOC*), *cytoskeleton-associated protein 2 like* (*CKAP2L*), *forkhead box I2* (*FOXI2*), *ALDH1L1 antisense RNA 2* (*ALDH1L1-AS2*), *IQ motif containing N* (*IQCN*), *transmembrane protein 211* (*TMEM211*), *anillin actin-binding protein* (*ANLN*), *abnormal spindle microtubule assembly* (*ASPM*), *PEAK family member 3* (*PEAK3*), *Interferon epsilon* (*IFNE*), *Chitinase-3-like protein 2* (*CHI3L2*), *Lymphokine-activated killer T-cell-originated protein kinase* (*PBK*), *V-set and immunoglobulin domain containing 4* (*VSIG4*), *long intergenic non-protein coding RNA 1411* (*LINC01411*), and *MIR31 host gene* (*MIR31HG*), were not picked by the pathway database, which indicates that our current understanding of OA pathogenesis is dreadfully inadequate.

**Figure 5:**
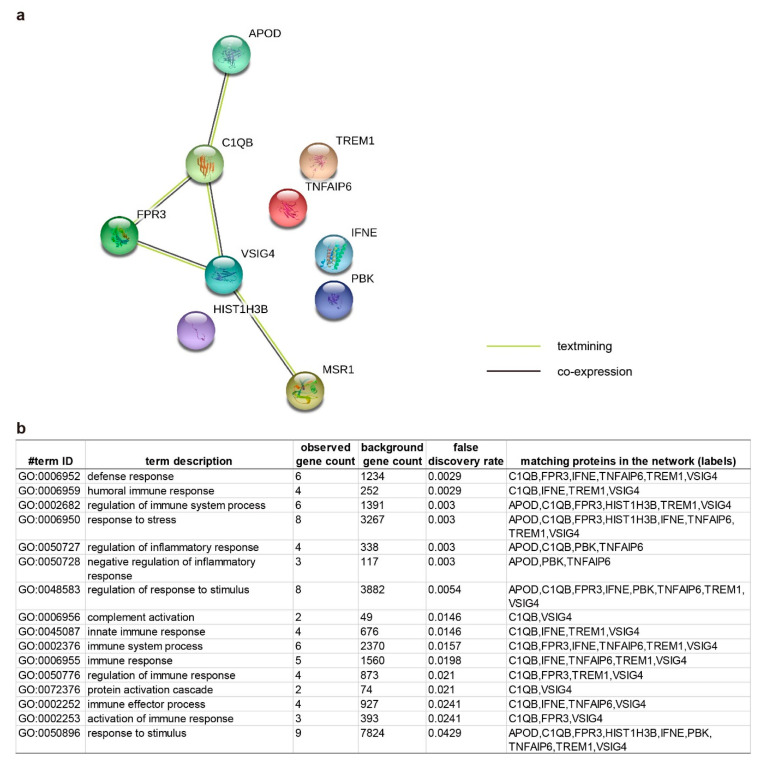
STRING database displayed the protein-protein interaction network among the 10 common differential expressed genes (DEGs) in cartilage and synovium that related to inflammation modulating. (**a**) the diagram generated from the STRING data based to demonstrate the potential interactions. (**b**) The list contains identified pathways with a false discovery rate (FDR) less than 0.05. *APOD*—*Apolipoprotein*; *C1QB*—*complement C1q subcomponent subunit B*; *FPR3*—*N-formyl peptide receptor 3*; *HIST1H3B*—*histone cluster 1 H3 family member b*; *IFNE*—*Interferon epsilon*; *MSR1*—*Macrophage scavenger receptor type I and II*; *PBK*—*Lymphokine-activated killer T-cell-originated protein kinase*; *TNFAIP6*—*Tumor necrosis factor-inducible gene 6 protein*; *TREM1*—*Triggering receptor expressed on myeloid cell 1*; *VSIG4*—*V-set and immunoglobulin domain containing 4*.

Since only 19 of 35 (54.3%) common OA-responsive DEGs in cartilage and synovium were picked by Reactome knowledgebase, manually align these 35 DEGs was achieved for further functional clustering. As inflammation is the primary and tissue shared event during OA, we first checked the DEGs that could relate to immune regulation. Based on the functional information collected in the Uniprot database [31] (Table S3), 10 DEGs, including *Apolipoprotein* (*APOD*), *complement C1q subcomponent subunit B* (*C1QB*), *N-formyl peptide receptor 3* (*FPR3*), *histone cluster 1 H3 family member b* (*HIST1H3B*), *IFNE*, *Macrophage scavenger receptor type I and II* (*MSR1*), *PBK*, *Tumor necrosis factor-inducible gene 6 protein* (*TNFAIP6*), *Triggering receptor expressed on myeloid cell 1* (*TREM1*), and *VSIG4*, were categorized to the inflammation-modulating group (Table 1). Protein-protein interactions among APOD, C1QB, FPR3, VSIG4, and MSR1 have also been assembled by the STRING networks [32]. These interactions, albeit weak (such as “textmining” and “co-expression”), were gathered in the biological processes related to “regulation of immune system process”, “response to stress”, “regulation of inflammatory response”, and “response to the stimulus” (Figure 5).

**Figure 6:**
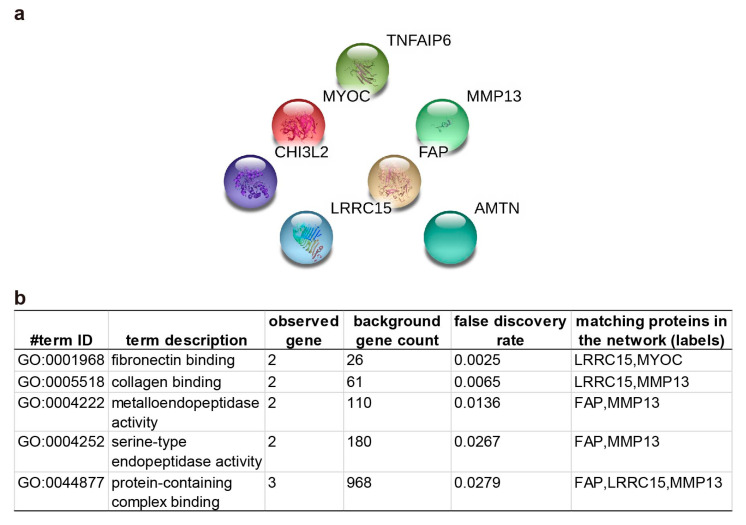
The clustering results from the STRING database by inputting 7 extracellular matrix related genes. (**a**) the diagram generated from the STRING data based to demonstrate the potential interactions. (**b**)The list contains identified pathways with a false discovery rate less than 0.05. *MYOC: Myocilin*, *AMTN: Amelotin*, *CHI3L2: Chitinase-3-like protein 2*, *FAP: Fibroblast activation protein alpha*, *LRRC15: Leucine-rich repeat-containing protein 15*, *MMP13: Matrix metallopeptidase-13*, *TNFAIP6: Tumor necrosis factor-inducible gene 6 protein.*

Additionally, 7 genes, including *MYOC*, *Amelotin* (*AMTN*), *CHI3L2*, *Prolyl endopeptidase FAP* (*Fibroblast activation protein alpha*; *FAP*), *LRRC15*, *MMP13*, and *TNFAIP6*, were grouped based on their functions related to “extracellular matrix (ECM) binding, formation, degradation” listed in the Uniprot database (Table 2). Although the STRING network could assemble no direct protein-protein interaction with statistical significance, several biological processes were detected, such as “fibronectin binding”, “collagen binding”, and “metalloendopeptidase activity” (Figure 6).

On the other hand, functions of the 3 DEGs whose OA-induced responses are different in synovium and cartilage were also examined. However, only *Phosphatase and actin regulator 3* (*PTACTR3*) could be found in the Uniprot database. As a protein expressed in the cell nucleus, PTACTR3 functions in “actin binding”, “protein phosphatase 1 binding” and “protein phosphatase inhibitor activity” (Table S4). No known OA-related function of the other two noncoding DEGs, *RNA, U12 small nuclear* (*RNU12*), and *RNA, U6 small nuclear 2* (*RNU6-2*) again highlights the lack of sufficient knowledge related to OA.

## 3. Discussion

It is no wonder that *matrix metallopeptidase 13* (*MMP13*), a broadly investigated critical OA-related gene [33,34], is one of the 35 DEGs that respond to OA the same trend in both cartilage and synovium, demonstrating the reliability of the current study. In addition to MMP13, some of these OA-responsive DEGs shared by cartilage and synovium have also been studied in the arena against arthritis, although their function may only be investigated solely in cartilage or synovium. For example, CHI3L2 (also known as YKL-39) has been identified as a biochemical marker for the OA progression in human cartilage since 2002 [35,36]. Functionally, CHI3HL2 has been shown to enhance the proliferation and type II collagen expression in ATDC5 mouse chondrogenic cells [37]. With the successful generation of polyclonal and monoclonal antibodies against CHI3L2, strong activation of the CHI3L2 was detected not only in human T lymphocyte cell lines and monocytes but in the synovial fluid of an OA patient [38]. More importantly, autoimmunity against CHI3L2 was also detected in OA patients [39]. However, its function on synovium tissue is still unknown.

On the other hand, as a transmembrane serine protease that is known to be associated with cell migration and cell invasiveness [40], FAP has been associated with arthritic synovium and cartilage. FAP-deficiency in hTNFtg mice led to less cartilage degradation [40], while highly expressed FAP has been detected in the rheumatoid synovium [41]. A radiolabeled anti–FAP antibody has even been used as a noninvasive strategy to monitor the course of collagen-induced arthritis in mice [41]. For the therapeutic drug development, targeted photodynamic therapy (tPDT) using the anti-FAP antibody 28H1 coupled to the photosensitizer IRDye700DX moderately delayed the collagen-induced arthritis development in mice [42]. Meanwhile, TREM-1 has also been identified as a biomarker of synovitis in RA [43] and predicting the therapeutic response to methotrexate in RA [44]. Inhibiting the expression of TREM-1 could suppress the chondrocyte injury induce by IL-1β in vitro [45], and ameliorate collagen-induced arthritis and protect bone and cartilage damage in vivo [46]. Although these published functional tests were predominantly archived in the RA scenario, especially with the RA synovial fibroblasts, our current study identified expression of FAP and TREM-1 significantly upregulated in both synovium and cartilage of OA patients, strongly suggesting that lowering FAP and TREM-1 might be beneficial for both RA and OA patients.

Besides, MSR1 is a multifunctional receptor expressed primarily on cells of the myeloid lineage [47]. It positively regulates the activation of macrophages and thus promoting the inflammation [48]. The genetic deficiency of *Msr1* decreased the incidence and severity of autoimmune arthritis in the K/BxN T cell receptor (TCR) transgenic mouse model [47]. It is well known that the immune system plays a critical role in OA pathogenesis, and previous studies have acknowledged that the application of immunomodulatory drugs is a potential avenue for OA treatments [49,50,51]. With the identification of MSR1 in the current study, we may further prove the immune processes’ participation within the OA joint and synovium [9].

TNFAIP6 (also known as TSG-6) is an upregulated OA-responsive DEG shared by cartilage and synovium. It is a multifunctional protein with anti-inflammatory and tissue-protective biopotencies [52]. It has been shown that intra-articular delivery of TNFAIP6 could reduce cartilage damage in a rat model of OA [53]. However, in the current study, the expression of TNFAIP6 is increased in both cartilage and synovium tissue during OA. These controversial findings could be explained by the article from Chou et al. published in 2018 [54]: TNFAIP6 was highly expressed in damaged articular and meniscal cartilage and cytokine-treated chondrocytes; functionally, TNFAIP6 impairs hyaluronan-aggrecan assembly, but TNFAIP6 mediated hyaluronan-heavy chain formation reduced this adverse effect. Thus, whether TNFAIP6 could be utilized for OA treatment or if it could worsen OA prognosis still needs further investigations.

Meanwhile, our finding is in line with the previous publication that 6-phosphofructo-2-kinase/fructose2,6-bisphosphatase 3 (PFKFB3) expression was down-regulated in human OA cartilage tissues [55]. PFKFB3 is a glycolytic regulator modulating glycolytic metabolism, alleviating endoplasmic reticulum stress, reducing caspase 3 activation, and promoting aggrecan and type II collagen expressions in human OA cartilage [55]. Interestingly, PFKFB3 expression was increased in RA patients’ synovial tissue but not in those of OA patients [56]. Moreover, inhibition of PFKFB3 suppresses the synovial inflammation and joint destruction in RA [56]. The distinct expression patterns and entirely opposite functions of PFKFB3 in OA and RA may set PFKFB3 as a biomarker for clinical differential diagnosis and may explain why some DMARDs are failing to improve OA.

Notably, we also identified multiple molecules that had not been correlated to OA. Nevertheless, their functions in chondrogenesis or inflammation make them as the novel targets for OA investigations in the future. For instance, APOD has been identified to be a downstream gene regulated by SRY-box transcription factor 9 (SOX9), an essential transcription factor for chondrocyte phenotype maintaining [57], in human chondrocytic cell line (SW1353) and primary human articular chondrocytes (hARCs) [58]. Particularly, *SOX9* downregulation in OA cartilage is followed by reduced expression of *APOD* [58]. Our finding is consistent with this publication that the expression of *APOD* lowered in OA cartilage tissue, and we also found that *APOD* is decreased in OA synovium. Interestingly, *SOX9* is not one of the 35 common OA-responsive DEGs shared by cartilage and synovium, suggesting that SOX9 may not play a critical role in OA’s synovium. Thus, APOD may participate in other SOX9-independent signaling pathways in the synovium tissue during OA progression. A recent study identified APOD as one of the novel molecular markers of human Th17 cells [59]. Although Th17 cells were initially investigated in RA due to their potency against autoimmune diseases [59], accumulating evidence suggests they are also increased in the OA [60]. Additionally, the high APOD protein level in the round ligament fat depot of severely obese women is associated with an improved inflammatory profile [61]. Thus, APOD may manage OA through chondrogenesis in articular cartilage and immune regulation in the synovium. Further functional investigations are necessary to verify this hypothesis.

Another example is C1QB, one of the C1Q genes which transcript component C1B predispose to RA [62]. The newly published paper demonstrated that primary hARCs express *C1QA*, *C1QB*, *C1QC*, and secret C1Q to the extracellular medium, while this expression is regulated by proinflammatory cytokines stimulations [63]. C1Q could bond to the chondrocytes in vitro, altering the expression of collagens [63]. C1Q can also bond to the cartilage matrix components, such as fibromodulin (FMOD), and activate the complement system to eliminate pathogens and damaged cells for tissue recovery and reconstruction [64]. Since the STRING database identified the biological process of “regulating the immune system process” the role of complement activation in both synovium and articular cartilage during OA might be worth more attention.

CDK1 is another commonly increased DEG in OA cartilage and synovium. As a member of the cyclin-dependent kinase family that plays a pivotal role in controlling the cell cycle [65], CDK1 is not only a hub node of the protein-protein interaction network of the 1,25-dihydroxy-vitamin D3 treated primary OA chondrocytes [66]; its expression is also related to the pathogenesis of RA [67]. It is interesting to find that CDK1 is one of the top 10 hub genes identified from the database of “osteoarthritic degenerative meniscal lesions” [65]. Thus, our current study supports the hypothesis that CDK1 plays an essential role in all three of cartilage, synovium, and meniscus. However, the “cell cycle” cluster has a broad range of effects on a diversity of biological events, and thus CDK1 may not be a unique biomarker for OA diagnosis or treatment.

FPR3 has two isoforms FPR1 and FPR2. The FPRs belong to the classical chemotactic G-protein-coupled receptor family that has recently been recognized to play critical roles in inflammation regulation in response to pathogen- or damage-associated chemotactic molecular patterns [68]. The FPRs agonists have been broadly investigated as a potential treatment strategy for various inflammation-related diseases [68]. Recently, the investigation of FPR agonists has extended to RA [69]. Thus, the anti-inflammation effect of FPR agonists might be utilized in OA targeting both the synovium and cartilage based on the finding in our research.

Our current finding is consistent with the previous studies demonstrating that the expression of KLF15 is significantly lower in chondrocytes from OA patients than from healthy subjects [70]. KLF15 is a transcriptional factor that could promote the chondrogenic differentiation of human mesenchymal stem cells by binding to the promoter of SOX9 and activating its expression [71]. Moreover, KLF15 could reduce the TNF-α-induced expression of MMP-3, a well-known cartilage-degrading enzyme [70]. On the other hand, KFL15 activation could negatively regulate inflammations in several cell types [72,73,74,75]. Thus, elevating KLF15 levels in OA may also achieve the dual effects of pro-chondrogenesis and anti-inflammation.

Last but not least, several identified genes have even not been correlated to joints so far. With the rapid development and advancement in the research fields, their OA correlations would be gradually established sooner or later. For example, amelotin (AMTN) is a novel secreted protein firstly identified in 2005 and was thought to be specific for ameloblasts [76]. Later on, most research about this protein was focused on its expression in gingival epithelial cells regulated by proinflammatory cytokines [77,78]. Since the current study distinguished *AMTN* as one of the upregulated genes in both synovium and cartilage in response to OA, its functional involvement in OA, particularly in inflammatory reactions, may be worth assessed. Additionally, KCNJ6 (also known as GIRK2) is a potassium channel regulator that holds potential as a pain-reducing target in OA patients [79,80].

It is well known that sex is an essential factor that significantly alters the gene expression in OA pathogenesis. In humans, the prevalence of OA is knowingly higher in women than men [4,6], while women typically present with worse symptoms, including more significant complaints of pain and disability [81,82]. Meanwhile, although OA is not an inevitable consequence of growing old, older age is the most significant risk factor for OA due to the accumulation of a diversity of OA inducers, such as joint injury, obesity, genetics, and anatomical factors that affect joint mechanics [83]. It is also possible that age-related cell senescence plays a critical role in promoting OA initiation and progression. Unfortunately, currently available transcriptome datasets are not collected to reflect the influence of ages, limiting our ability to draw the “blueprint” of age-related OA gene expression. Clearly, it is an urgent task to conquer in future investigations. On the other hand, as the current study aims to identify the potential unisex markers in OA synovium and cartilage and to get the insight into novel OA therapies effective in both male and female OA patients with all ages, we believe that the identified targets mentioned above are valid for the new generation of DMOAD developing.

## 4. Materials and Methods

SRA files of healthy and OA human knee cartilage (GEO accession number: GSE114007) and health and OA synovium (GEO accession number: GSE89408) RNA-seq data were downloaded from https://www.ncbi.nlm.nih.gov/sra. Data analyses were performed on the Galaxy platform (UseGalaxy.org, [84]). The FASTQC RNA-seq reads were aligned to the human genome (GRCh38) using HISAT2 aligner (Galaxy v. 2.1.0+galaxy 5) with default parameters [85]. Samples with an overall alignment rate >75% were used for further analysis. Raw counts of sequencing read for the feature of genes were extracted by featureCounts (Galaxy v. 1.6.4+galaxy1) [86]. Then, the limma package (Galaxy v. 3.38.3+galaxy3) was used to identify DEGs with its voom method [87,88]. Expressed genes were selected as their counts per million (CPM) value not less than 1 in at least two samples across the entire experiment, while lowly expressed genes were removed for the flowing analyses. Quasi-likelihood F-tests (ANOVA-like analysis) were achieved to identify DEGs [89]. Genes with fold change (FC) more than 2 and false discovery rate (FDR) less than 0.01 were assigned as DEGs. Heatmap, multidimensional scaling (MDS), principal component analysis (PCA), and the Venn diagram were conducted in R (v. 3.6.3) [90] with packages pheatmap (v. 1.0.12), vegan (v. 2.5-6), ggplot2 (v. 3.3.0), and VennDiagram (v. 1.6.20). Pathway enrichment of identified DEGs was firstly performed against the Reactome knowledgebase [30]. In addition, the summary of the known biofunctions for these genes was searched in the Uniprot database [31] for manually functional annotation. The STRING network [32] was also utilized for the protein-protein association and interaction assembling.

## 5. Conclusions

In summary, our current study identified several novel genes as the potential biomarkers or treatment targets for OA, which will benefit both synovium and cartilage. In combination with the excellent works published by worldwide researchers, we hope our work could pave the path for developing the new generation of DMOADs.

## Figures and Tables

**Table 1:** Ten common DEGs related to “inflammation modulating”.

Symbol	Gene Name	Healthy vs. OA				Expression	Function in Uniprot
		Synovium		Cartilage			
		logFC	*p* Value	logFC	*p* Value		
*APOD*	*Apolipoprotein D*	−2.30916619	0.025415	−3.64045	0.000375	Extracellular region or secreted	Aging, angiogenesis, brain development, glucose metabolic process, lipid metabolic process, negative regulation of cytokine production involved in inflammatory response, negative regulation of focal adhesion assembly, negative regulation of lipoprotein lipid oxidation, negative regulation of monocyte chemotactic protein-1 production, negative regulation of platelet-derived growth factor receptor signaling pathway, negative regulation of protein import into nucleus, negative regulation of smooth muscle cell-matrix adhesion, negative regulation of smooth muscle cell proliferation, negative regulation of T cell migration, peripheral nervous system axon regeneration, response to axon injury, response to drug, response to reactive oxygen species, tissue regeneration
*C1QB*	*Complement C1q subcomponent subunit B*	1.71705429	0.036863	2.74003	0.042712	Extracellular region or secreted	Complement activation, classical pathway; innate immune response, inner ear development, regulation of complement activation, synapse pruning
*FPR3*	*N-formyl peptide receptor 3*	2.00718964	0.004814	2.587989	0.045465	Plasma membrane	Chemotaxis, complement receptor mediated signaling pathway, G protein-coupled receptor signaling pathway, inflammatory response, phospholipase C-activating G protein-coupled receptor signaling pathway, positive regulation of cytosolic calcium ion concentration, signal transduction
*HIST1H3B*	*Histone cluster 1 H3 family member b*	1.89596286	0.033674	2.685807	0.025722	Nucleus	Blood coagulation, cellular protein metabolic process, chromatin organization, chromatin silencing at rDNA, DNA replication-dependent nucleosome assembly, interleukin-7 mediated signaling pathway, negative regulation of gene expression, epigenetic, nucleosome assembly, regulation of gene silencing by miRNA, regulation of megakaryocyte differentiation, telomere organization
*IFNE*	*Interferon epsilon*	2.74670721	0.001932	3.545149	0.000413	Extracellular region or secreted	Adaptive immune response, B cell differentiation, B cell proliferation, cytokine-mediated signaling pathway, defense response to bacterium, defense response to virus, humoral immune response, natural killer cell activation involved in immune response, positive regulation of peptidyl-serine phosphorylation of STAT protein, response to exogenous dsRNA, T cell activation involved in immune response
*MSR1*	*Macrophage scavenger receptor types I and II*	2.00838663	0.011941	3.360986	0.015031	-	Amyloid-beta clearance, cholesterol transport, negative regulation of gene expression, phagocytosis, engulfment, plasma lipoprotein particle clearance, positive regulation of cholesterol storage, positive regulation of macrophage derived foam cell differentiation, receptor-mediated endocytosis
*PBK*	*Lymphokine-activated killer T-cell-originated protein kinase*	1.94190515	0.035765	2.802857	0.011073	Nucleus	Cellular response to UV, mitotic cell cycle, negative regulation of inflammatory response, negative regulation of proteasomal ubiquitin-dependent protein catabolic process, negative regulation of stress-activated MAPK cascade
*TNFAIP6*	*Tumor necrosis factor-inducible gene 6 protein*	2.42594431	0.004714	4.335193	0.000138	Extracellular region or secreted	Hyaluronic acid binding; cell adhesion, cell-cell signaling, inflammatory response, negative regulation of inflammatory response, neutrophil degranulation, ovulation, positive regulation of cell migration, signal transduction
*TREM1*	*Triggering receptor expressed on myeloid cells 1*	1.77423941	0.042306	4.671057	3.93 × 10^−5^	Extracellular region or secreted, Plasma membrane	Acute inflammatory response, humoral immune response, innate immune response, intracellular signal transduction, leukocyte migration, regulation of immune response
*VSIG4*	*V-set and immunoglobulin domain-containing protein 4*	1.90947583	0.019372	3.174618	0.024179	-	Negative regulation of complement activation, alternative pathway; negative regulation of interleukin-2 production, negative regulation of macrophage activation, negative regulation of T cell proliferation

OA—osteoarthritis; logFC—log fold change; rDNA—Recombinant DNA; miRNA—microRNA; STAT—signal transducer and activator of transcription; dsRNA—Double-stranded RNA; UV—Ultraviolet; MAPK—mitogen-activated protein kinase.

**Table 2:** Seven common DEGs related to “extracellular matrix (ECM) binding, formation, degradation.”

Symbol	Gene Name	Healthy vs. OA				Expression	Function in Uniprot
		Synovium		Cartilage			
		logFC	*p* Value	logFC	*p* Value		
*MYOC*	*Myocilin*	−3.2029017	0.00068	−4.11544	0.002817	Extracellular region or secreted, Golgi apparatus, mitochondrion, rough endoplasmic reticulum	Bone development, clustering of voltage-gated sodium channels, ERBB2-ERBB3 signaling pathway, myelination in peripheral nervous system, negative regulation of cell-matrix adhesion, negative regulation of Rho protein signal transduction, negative regulation of stress fiber assembly, neuron projection development, non-canonical Wnt signaling pathway via JNK cascade, osteoblast differentiation, positive regulation of cell migration, positive regulation of focal adhesion assembly, positive regulation of mitochondrial depolarization, positive regulation of phosphatidylinositol 3-kinase signaling, positive regulation of protein kinase B signaling, positive regulation of stress fiber assembly, positive regulation of substrate adhesion-dependent cell spreading, regulation of MAPK cascade, skeletal muscle hypertrophy
*AMTN*	*Amelotin*	3.91241415	0.002914	5.414278	0.000259	Extracellular region or secreted	Biomineral tissue development, cell adhesion, cellular protein metabolic process, odontogenesis of dentin-containing tooth, positive regulation of biomineral tissue development, positive regulation of enamel mineralization, post-translational protein modification
*CHI3L2*	*Chitinase-3-like protein 2*	2.17945593	0.023414	3.223409	0.000902	Extracellular region or secreted	Carbohydrate metabolic process, chitin catabolic process
*FAP*	*Prolyl endopeptidase FAP/Fibroblast activation protein alpha*	1.68745632	0.044592	2.264122	0.013885	Extracellular region or secreted, plasma membrane	Angiogenesis, cell adhesion, endothelial cell migration, melanocyte apoptotic process, melanocyte proliferation, mitotic cell cycle arrest, negative regulation of cell proliferation involved in contact inhibition, negative regulation of extracellular matrix disassembly, negative regulation of extracellular matrix organization, positive regulation of cell cycle arrest, positive regulation of execution phase of apoptosis, proteolysis, proteolysis involved in cellular protein catabolic process, regulation of collagen catabolic process, regulation of fibrinolysis
*LRRC15*	*Leucine-rich repeat-containing protein 15*	2.10204668	0.016016	5.236099	2.11 × 10^−5^	Extracellular region or secreted	Collagen binding, fibronectin binding, laminin binding; negative regulation of protein localization to plasma membrane, positive regulation of cell migration, receptor-mediated virion attachment to host cell
*MMP13*	*Matrix metallopeptidase-13/Collagenase 3*	3.48223529	7.54 × 10^−5^	3.194238	0.001586	Extracellular region or secreted	Bone mineralization, bone morphogenesis, cellular protein metabolic process, collagen catabolic process, endochondral ossification, extracellular matrix disassembly, extracellular matrix organization, growth plate cartilage development, proteolysis, response to amyloid-beta
*TNFAIP6*	*Tumor necrosis factor-inducible gene 6 protein*	2.42594431	0.004714	4.335193	0.000138	Extracellular region or secreted	Hyaluronic acid binding; cell adhesion, cell-cell signaling, inflammatory response, negative regulation of inflammatory response, neutrophil degranulation, ovulation, positive regulation of cell migration, signal transduction

OA—osteoarthritis; logFC—log fold change; ERBB—epidermal growth factor; Wnt—Wingless-related integration site; JNK-c—Jun N-terminal kinases; MAPK—mitogen-activated protein kinase.

## Supplementary Materials

Supplementary materials can be found at https://www.mdpi.com/1422-0067/21/17/6033/s1. Table S1. The information of the samples included in the current study; Table S2. The list of the identified pathways from the Reactome database; Table S3. The biological processes described in the Uniprot database for the genes with the same expression trends in response to OA; Table S4. The biological processes described in the Uniprot database for the genes with different expression trends in response to OA.
